# Supplementary material for: Dairy Consumption at Breakfast among Southeast Asian Children: Associations with Nutrient Intake from the South East Asian Nutrition Surveys II (SEANUTS II)
Source: Nutrients. 2024 Sep 24;16(19):3229. doi: 10.3390/nu16193229 (PMC11478093; doi:10.3390/nu16193229)
Supplement: Supplementary file 1 [file nutrients-16-03229-s001.zip › nutrients-3191780-supplementary.pdf]

SUPPLEMENTARY MATERIAL

RESULTS

Energy and nutrient intake at breakfast between children consuming versus not consuming dairy at breakfast according to the 24-hour dietary recall stratified by age groups

Tables S1–S4 present the energy and nutrient intake at breakfast among breakfast consuming children, stratified by age group in Malaysia, Thailand, Indonesia, and Vietnam, respectively.

Table S1. Energy and nutrient intake at breakfast in children consuming versus not consuming dairy at breakfast stratified per age group in Malaysia using the 24-hour dietary recall.

	**2 to 3 years old**		**4 to 6 years old**		**7 to 12 years old**	
	**Not consumed dairy (n = 106)**	**Consumed dairy (n = 216)**	**Not consumed dairy (n = 406)**	**Consumed dairy (n = 345)**	**Not consumed dairy (n = 998)**	**Consumed dairy (n = 319)**
Energy, kcal	222 (144)	223 (155)	235 (181)	221 (180)	280 (244)	286 (208)
Protein, g	6.4 (7.8)	6.8 (5.4)	6.8 (7.5)	7.0 (6.0)	7.8 (10.0)	9.1 (8.0) **
Carbohydrates, g	30.4 (21.5)	29.5 (22.3)	33.6 (25.9)	30.3 (22.2) *	38.8 (32.9)	37.0 (30.4)
Fat, g	7.0 (8.3)	7.4 (6.1)	7.4 (8.06)	7.0 (6.9)	8.7 (10.2)	8.9 (9.5)
Vitamin A, μg RE	84 (116)	147 (93) ***	98 (135)	160 (111) ***	115 (159)	205 (185) ***
β-carotene, μg[1]	8.0 (52.7)	58.8 (51.3) ***	7.2 (33.4)	40.4 (45.6) ***	5.5 (30.0)	22.3 (47.1) ***
Vitamin B_1, mg	0.11 (0.20)	0.22 (0.22) ***	0.16 (0.25)	0.26 (0.28) ***	0.19 (0.29)	0.45 (0.51) ***
Vitamin B_2, mg	0.18 (0.25)	0.33 (0.26) ***	0.25 (0.28)	0.37 (0.33) ***	0.26 (0.30) ***	0.48 (0.42)
Vitamin B_3, mg	1.41 (1.83)	2.20 (1.60) ***	2.11 (2.47)	2.62 (2.28) ***	2.46 (2.81)	3.54 (3.71) ***
Vitamin B_{12}, μg	0.11 (0.40)	0.50 (0.38) ***	0.21 (0.59)	0.48 (0.44) ***	0.22 (0.63)	0.50 (0.55) ***
Vitamin C, mg	1.1 (7.3)	19.8 (19.3) ***	3.77 (9.24)	17.7 (18.0) ***	5.5 (11.5)	14.6 (16.5) ***
Vitamin D, μg	0.07 (0.94)	2.24 (1.87) ***	0.40 (1.20)	2.57 (2.38) ***	0.45 (1.24)	2.41 (3.06) ***
Calcium, mg	57 (76)	227 (122) ***	75 (106)	256 (143) ***	93 (130)	276 (218) ***
Iron, mg	1.55 (1.80)	2.76 (1.72) ***	2.00 (2.02)	3.17 (2.41) ***	2.32 (2.46)	3.34 (3.42) ***
Sodium, mg	284 (363)	135 (173) ***	284 (429)	138 (184) ***	289 (481)	220 (305) ***
Potassium, mg	113 (131)	253 (134) ***	133 (148)	272 (171) ***	161 (178)	276 (200) ***
Phosphorus, mg	81 (87)	168 (87) ***	96 (91)	186 (116) ***	118 (120)	210 (154) ***

Data presented as median (IQR). Data was analyzed with Wilcoxon's rank-sum tests. Significant differences from children who did not consume dairy are indicated as *** p <0.001, ** p <0.01, * p <0.05. [1] Outlier removed in children aged 7 to 12 years: Not consumed dairy (n = 831), Consumed dairy (n = 292).

Table S2. Energy and nutrient intake at breakfast in children consuming versus not consuming dairy at breakfast stratified per age group in Thailand using the 24-hour dietary recall.

	**2 to 3 years old**		**4 to 6 years old**		**7 to 12 years old**	
	**Not consumed dairy (n = 398)**	**Consumed dairy (n = 324)**	**Not consumed dairy (n = 635)**	**Consumed dairy (n = 207)**	**Not consumed dairy (n = 884)**	**Consumed dairy (n = 161)**
Energy, kcal[1]	248 (185)	320 (197) ***	317 (209)	392 (223) ***	382 (275)	419 (229)
Protein, g	10.2 (7.5)	15.3 (9.6) ***	12.7 (9.3)	16.9 (10.4) ***	14.5 (12.0)	17.4 (14.9) **
Carbohydrates, g	32.1 (25.3)	36.2 (25.8) **	40.4 (32.5)	43.5 (27.5)	51.3 (39.7)	50.2 (36.9)
Fat, g	6.9 (8.9)	12.2 (8.8) ***	9.4 (10.6)	15.3 (13.2) ***	10.6 (13.9)	15.1 (12.8) ***
Fiber, g	0.54 (0.74)	0.97 (1.55) ***	0.60 (0.74)	1.32 (1.78) ***	0.78 (1.01)	1.52 (1.51) ***
Vitamin A, µg RAE	44 (112)	131 (147) ***	39 (142)	147 (136) ***	20 (126)	91 (126) ***
Vitamin B_1, mg	0.11 (0.14)	0.20 (0.25) ***	0.15 (0.32)	0.27 (0.55) ***	0.18 (0.29)	0.24 (0.42) **
Vitamin B_2, mg	0.14 (0.21)	0.47 (0.23) ***	0.20 (0.26)	0.52 (0.27) ***	0.21 (0.26)	0.49 (0.25) ***
Vitamin B_3, mg	1.63 (2.11)	2.04 (2.74) ***	2.43 (3.61)	2.53 (3.38)	3.16 (3.46)	2.76 (4.67)
Vitamin B_{12}, µg	0.32 (0.58)	0.71 (0.83) ***	0.38 (0.60)	0.83 (0.85) ***	0.37 (0.59)	0.87 (0.74) ***
Vitamin C, mg	0.5 (3.3)	3.0 (11.4) ***	0.8 (7.5)	1.1 (7.1)	0.8 (5.6)	1.0 (6.0)
Vitamin D, µg	0.44 (1.25)	1.90 (1.52) ***	0.47 (1.32)	2.07 (1.49) ***	0.29 (1.29)	1.98 (1.08) ***
Calcium, mg	32 (52)	227 (93) ***	36 (74)	242 (85) ***	34 (61)	229 (75) ***
Iron, mg	1.13 (1.04)	1.56 (2.23) ***	1.44 (1.30)	1.40 (1.62)	1.65 (1.45)	1.39 (1.44) **
Zinc, mg	0.81 (0.71)	1.15 (1.21) ***	1.07 (0.86)	1.09 (1.16)	1.25 (1.21)	1.13 (1.39)
Magnesium, mg[2]	13.4 (14.0)	16.6 (16.9) *	19.4 (22.3)	19.0 (20.09)	21.8 (22.8)	24.0 (26.4)
Sodium, mg	336 (437)	352 (434)	423 (510)	386 (409)	479 (597)	439 (592)
Potassium, mg	156 (138)	257 (255) ***	190 (164)	246 (260) ***	220 (200)	280 (278) **
Phosphorus, mg	100 (103)	176 (141) ***	129 (120)	189 (179) ***	138 (130)	232 (176) ***

Data presented as median (IQR). Data was analyzed with Wilcoxon's rank-sum tests. Significant differences from children who did not consume dairy are indicated as *** p <0.001, ** p <0.01, * p <0.05. [1] Outlier removed in children aged 7-12 years: Not consumed dairy (n = 858), Consumed dairy (n = 153). [2] Outlier removed in children aged 2 to 3 years: Not consumed dairy (n = 370), Consumed dairy (n = 313).

Table S3. Energy and nutrient intake at breakfast in children consuming versus not consuming dairy at breakfast stratified per age group in Indonesia using the 24-hour dietary recall.

	**2 to 3 years old**		**4 to 6 years old**		**7 to 12 years old**	
	**Not consumed dairy (n = 440)**	**Consumed dairy (n = 187)**	**Not consumed dairy (n = 428)**	**Consumed dairy (n = 93)**	**Not consumed dairy (n = 975)**	**Consumed dairy (n = 65)**
Energy, kcal	200 (133)	274 (176) ***	232 (164)	350 (146) ***	255 (166)	329 (237) ***
Protein, g	7.1 (5.8)	9.1 (6.6) ***	8.3 (6.8)	12.9 (4.9) ***	8.5 (6.3)	11.7 (9.9) ***

Carbohydrates, g	22.4 (17.5)	33.2 (20.1) ***	28.3 (19.9)	42.2 (22.2) ***	29.3 (24.4)	43.6 (30.4) ***
Fat, g	7.8 (9.8)	10.1 (9.5) ***	11.0 (9.8)	15.3 (7.6) ***	11.3 (9.8)	12.6 (10.9) *
Fiber, g	0.48 (0.68)	0.90 (0.91) ***	0.47 (0.91)	0.90 (1.10) ***	0.60 (0.94)	1.12 (1.90) *
Vitamin A, μg RE	35 (89)	137 (118) ***	35 (93)	155 (106) ***	35 (61)	148 (90) ***
Vitamin B_1, mg	0.06 (0.06)	0.18 (0.15) ***	0.07 (0.08)	0.28 (0.27) ***	0.07 (0.08)	0.33 (0.48) ***
Vitamin B_2, mg	0.10 (0.15)	0.28 (0.24) ***	0.17 (0.16)	0.42 (0.25) ***	0.15 (0.15)	0.48 (0.34) ***
Vitamin B_{12}, μg	0.35 (0.59)	0.58 (0.69) ***	0.53 (0.68)	0.92 (0.74) ***	0.45 (0.63)	0.72 (0.96) ***
Vitamin C, mg	0.1 (2.3)	12.0 (10.3) ***	0.1 (1.6)	13.6 (11.9) ***	0.0 (1.1)	19.8 (31.5) ***
Vitamin D, μg	0.22 (0.65)	2.37 (1.61) ***	0.24 (0.70)	2.90 (2.21) ***	0.27 (0.65)	3.94 (2.09) ***
Calcium, mg	46 (51)	189 (138) ***	58 (58)	279 (186) ***	57 (61)	355 (154) ***
Iron, mg	1.14 (1.34)	2.27 (1.71) ***	1.59 (1.46)	3.60 (2.04) ***	1.60 (1.47)	3.96 (3.75) ***
Zinc, mg	0.80 (0.54)	1.66 (1.17) ***	0.91 (0.66)	2.14 (1.25) ***	0.94 (0.74)	2.31 (1.64) ***
Choline, mg	27 (140)	26 (31)	34 (139)	86 (134) ***	36 (136)	48 (75)
DHA, mg	3.9 (19.7)	9.1 (16.3) ***	2.9 (19.7)	19.7 (21.6) ***	3.1 (19.7)	0.5 (19.7)

Data presented as median (IQR). Data was analyzed with Wilcoxon's rank-sum tests. Significant differences from children who did not consume dairy are indicated as *** p <0.001, * p <0.05.

Table S4. Energy and nutrient intake at breakfast in children consuming versus not consuming dairy at breakfast stratified per age group in Vietnam using the 24-hour dietary recall.

	**2 to 3 years old**		**4 to 6 years old**		**7 to 12 years old**	
	**Not consumed dairy (n = 493)**	**Consumed dairy (n = 169)**	**Not consumed dairy (n = 563)**	**Consumed dairy (n = 170)**	**Not consumed dairy (n = 1345)**	**Consumed dairy (n = 169)**
Energy, kcal	155 (121)	214 (122) ***	192 (144)	264 (168) ***	233 (164)	324 (166) ***
Protein, g	6.0 (5.2)	8.7 (5.3) ***	7.3 (6.1)	9.4 (6.6) ***	8.5 (7.8)	12.6 (7.6) ***
Carbohydrates, g	23.8 (22.0)	27.0 (20.0)	29.3 (24.7)	34.5 (21.8) *	35.8 (26.9)	39.5 (22.7)
Fat, g	2.5 (4.5)	6.7 (4.8) ***	3.9 (6.1)	8.3 (6.9) ***	4.7 (6.7)	11.0 (6.8) ***
Vitamin A, μg RAE	1.6 (47.6)	82.6 (62.2) ***	1.2 (37.7)	86.6 (61.2) ***	2.4 (49.4)	93.9 (45.6) ***
Vitamin B_{12}, μg	0.07 (0.29)	0.48 (0.29) ***	0.13 (0.40)	0.68 (0.39) ***	0.14 (0.41)	0.79 (0.27) ***
Vitamin C, mg	0.07 (1.14)	1.10 (1.68) ***	0.01 (0.43)	1.10 (1.50) ***	0.00 (0.55)	1.80 (1.10) ***
Vitamin D, μg	0.05 (0.18)	1.65 (1.44) ***	0.08 (0.26)	1.65 (0.94) ***	0.06 (0.28)	1.80 (0.70) ***
Calcium, mg	25 (34)	154 (69) ***	25 (32)	157 (85) ***	28 (34)	224 (82) ***
Iron, mg[1]	0.88 (0.79)	1.00 (1.32) *	1.07 (1.08)	1.08 (1.20)	1.25 (1.07)	1.37 (1.14)
Zinc, mg	0.88 (0.85)	1.17 (0.94) ***	0.97 (1.12)	1.27 (1.01) ***	1.17 (1.24)	1.51 (1.40) ***

Data presented as median (IQR). Data was analyzed with Wilcoxon's rank-sum tests. Significant differences from children who did not consume dairy are indicated as *** p <0.001, * p <0.05. [1] Outlier removed in children aged 2 to 3 years: Not consumed dairy (n = 453), Consumed dairy (n = 166).

Total daily energy and nutrient intake between children consuming versus not consuming dairy at breakfast according to the 24-hour dietary recall stratified by age groups

Table S1. Total daily energy and nutrient intake in children consuming versus not consuming dairy at breakfast stratified per age group in Malaysia using the 24-hour dietary recall.

	**2 to 3 years old**		**4 to 6 years old**		**7 to 12 years old**	
	**Not consumed dairy (n = 106)**	**Consumed dairy (n = 216)**	**Not consumed dairy (n = 406)**	**Consumed dairy (n = 345)**	**Not consumed dairy (n = 998)**	**Consumed dairy (n = 319)**
Energy, kcal	1190 (463)	1240 (433)	1240 (543)	1370 (559) ***	1470 (653)	1510 (535)
Protein, g	42.3 (18.2)	45.8 (17.9) *	44.5 (23.0)	50.9 (22.5) ***	53.4 (27.9)	55.2 (27.1)
Carbohydrates, g	163 (79)	161 (61)	164 (77)	183 (79) ***	199 (93)	203 (83)
Fat, g	39.4 (18.6)	43.3 (17.7)	41.9 (24.6)	46.7 (24.3) **	48.5 (28.6)	52.7 (23.8) *
Vitamin A, µg RE	513 (398)	719 (360) ***	548 (414)	671 (469) ***	595 (539)	814 (606) ***
β-carotene, µg	591 (1140)	767 (1130) **	461 (974)	599 (1150) **	728 (1590)	1040 (2100) **
Vitamin B_1, mg	0.79 (0.62)	1.07 (0.63) ***	0.85 (0.63)	1.18 (0.80) ***	0.96 (0.77)	1.34 (0.84) ***
Vitamin B_2, mg	1.06 (0.68)	1.46 (0.74) ***	1.18 (0.84)	1.51 (0.91) ***	1.20 (0.83)	1.58 (0.98) ***
Vitamin B_3, mg	9.1 (6.2)	10.9 (5.0) ***	9.6 (6.9)	12.3 (7.2) ***	10.9 (7.5)	12.6 (8.3) ***
Vitamin B_{12}, µg	1.79 (1.97)	2.71 (2.19) ***	2.14 (2.39)	2.85 (2.38) ***	2.25 (2.71)	2.64 (2.74) **
Vitamin C, mg	53.1 (56.7)	96.1 (84.2) ***	39.3 (48.4)	73.0 (86.8) ***	45.9 (68.5)	68.0 (83.3) ***
Vitamin D, µg	4.00 (5.86)	8.14 (6.02) ***	3.31 (4.53)	6.98 (7.26) ***	3.05 (4.01)	5.23 (5.74) ***
Calcium, mg	504 (310)	846 (431) ***	441 (342)	755 (486) ***	475 (362)	728 (414) ***
Iron, mg	9.0 (5.2)	12.3 (5.4) ***	9.7 (5.6)	12.8 (6.8) ***	10.6 (7.0)	12.3 (7.5) ***
Sodium, mg	1310 (727)	1140 (830)	1540 (1030)	1540 (927)	1840 (1400)	1780 (1130)
Potassium, mg	883 (506)	1230 (585) ***	884 (499)	1170 (692) ***	976 (606)	1170 (580) ***
Phosphorus, mg	572 (308)	770 (325) ***	576 (365)	807 (413) ***	650 (408)	791 (403) ***

Data presented as median (IQR). Data was analyzed with Wilcoxon's rank-sum tests. Significant differences from children who did not consume dairy are indicated as *** p <0.001, ** p <0.01, * p <0.05.

Table S2. Total daily energy and nutrient intake in children consuming versus not consuming dairy at breakfast stratified per age group in Thailand using the 24-hour dietary recall.

	**2 to 3 years old**		**4 to 6 years old**		**7 to 12 years old**	
	**Not consumed dairy (n = 398)**	**Consumed dairy (n = 324)**	**Not consumed dairy (n = 635)**	**Consumed dairy (n = 207)**	**Not consumed dairy (n = 884)**	**Consumed dairy (n = 161)**
Energy, kcal	1260 (462)	1320 (456) *	1450 (596)	1460 (608)	1670 (715)	1700 (650)
Protein, g[1]	47.3 (20.3)	52.8 (21.7) ***	52.7 (24.4)	56.4 (26.4) *	59.4 (30.8)	64.3 (26.0) *
Carbohydrates, g	156 (68)	147 (69)	181 (86)	178 (79)	223 (99)	222 (91)
Fat, g	45.9 (26.0)	52.3 (24.2) ***	51.5 (29.2)	54.7 (30.4) **	54.6 (35.6)	60.3 (32.9) **

Fiber, g	4.89 (3.87)	5.11 (4.87)	5.36 (3.57)	5.85 (4.49) *	6.21 (4.24)	7.09 (4.78) **
Vitamin A, µg RAE	357 (308)	481 (330) ***	321 (298)	419 (280) ***	262 (275)	335 (368) ***
Vitamin B_1, mg	0.73 (0.60)	0.75 (0.66)	0.83 (0.74)	0.93 (0.92) ***	0.84 (0.75)	1.00 (0.84) **
Vitamin B_2, mg	1.25 (0.82)	1.69 (0.76) ***	1.22 (0.80)	1.59 (0.71) ***	1.02 (0.71)	1.44 (0.78) ***
Vitamin B_3, mg	7.23 (5.73)	7.70 (7.21)	9.51 (6.95)	9.74 (7.40)	11.4 (7.46)	11.9 (7.72)
Vitamin B_{12}, µg	1.80 (1.89)	2.26 (2.41) ***	1.95 (1.68)	2.50 (2.00) ***	1.87 (1.88)	2.48 (2.00) **
Vitamin C, mg	19.9 (32.6)	22.4 (38.9)	21.3 (25.5)	19.3 (25.0)	22.8 (29.4)	19.3 (22.4)
Vitamin D, µg	4.87 (3.92)	7.02 (4.99) ***	4.13 (3.65)	6.33 (3.42) ***	3.18 (3.25)	5.09 (3.14) ***
Calcium, mg	541 (346)	789 (441) ***	454 (315)	672 (373) ***	334 (285)	567 (273) ***
Iron, mg	5.12 (3.84)	5.39 (6.55)	5.99 (3.61)	5.72 (4.61)	7.13 (4.30)	7.11 (4.76)
Zinc, mg	3.23 (2.06)	3.68 (3.26) **	3.96 (2.02)	3.97 (2.16)	4.71 (2.68)	4.62 (2.51)
Magnesium, mg	72.6 (49.6)	69.0 (51.9)	89.2 (60.4)	83.3 (48.5) *	102 (66.7)	108 (61.2)
Sodium, mg	1530 (1020)	1480 (1060)	1910 (1190)	1960 (1240)	2390 (1660)	2200 (1540)
Potassium, mg	891 (592)	1050 (818) **	1000 (618)	1130 (711) **	1140 (696)	1250 (610)
Phosphorus, mg	501 (342)	626 (476) ***	583 (341)	642 (405) **	622 (400)	728 (364) ***

Data presented as median (IQR). Data was analyzed with Wilcoxon's rank-sum tests. Significant differences from children who did not consume dairy are indicated as *** p <0.001, ** p <0.01, * p <0.05. [1] Outlier removed in children aged 7 to 12 years: Not consumed dairy (n = 860), Consumed dairy (n = 151).

Table S3. Total daily energy and nutrient intake in children consuming versus not consuming dairy at breakfast stratified per age group in Indonesia using the 24-hour dietary recall.

	**2 to 3 years old**		**4 to 6 years old**		**4 to 6 years old**	
	**Not consumed dairy (n = 440)**	**Consumed dairy (n = 187)**	**Not consumed dairy (n = 428)**	**Consumed dairy (n = 93)**	**Not consumed dairy (n = 975)**	**Consumed dairy (n = 65)**
Energy, kcal	911 (520)	1180 (545) ***	1060 (545)	1450 (678) ***	1240 (588)	1480 (530) ***
Protein, g	28.0 (16.8)	37.3 (17.8) ***	32.5 (18.6)	46.0 (19.1) ***	37.9 (20.6)	47.7 (24.8) ***
Carbohydrates, g	117 (67)	152 (69) ***	130 (72)	186 (77) ***	157 (81)	190 (93) ***
Fat, g	34.8 (25.6)	43.8 (22.7) ***	42.7 (26.9)	56.4 (31.9) ***	49.3 (29.6)	58.7 (30.1) **
Fiber, g	3.39 (3.29)	4.79 (4.07) ***	3.54 (3.92)	5.41 (4.42) ***	4.59 (4.53)	4.89 (5.87)
Vitamin A, µg RE	352 (374)	652 (494) ***	347 (503)	638 (460) ***	288 (356)	444 (378) ***
Vitamin B_1, mg	0.50 (0.46)	0.82 (0.48) ***	0.55 (0.58)	1.06 (0.66) ***	0.47 (0.44)	0.95 (0.71) ***
Vitamin B_2, mg	0.68 (0.54)	1.22 (0.74) ***	0.75 (0.70)	1.57 (0.83) ***	0.68 (0.53)	1.28 (0.86) ***
Vitamin B_{12}, µg	1.97 (1.97)	2.76 (2.31) ***	2.22 (2.33)	3.72 (2.55) ***	2.12 (2.11)	3.04 (1.88) ***
Vitamin C, mg	13.4 (30.1)	53.7 (48.4) ***	10.4 (28.5)	54.5 (49.5) ***	10.6 (22.8)	34.0 (66.7) ***
Vitamin D, µg	2.70 (4.02)	8.96 (7.05) ***	2.45 (3.89)	9.43 (8.30) ***	1.54 (2.48)	5.62 (4.31) ***
Calcium, mg	342 (326)	781 (476) ***	316 (322)	1010 (596) ***	289 (270)	654 (480) ***
Iron, mg	5.3 (3.9)	9.5 (6.4) ***	6.1 (4.3)	12.7 (7.3) ***	7.0 (4.3)	11.0 (6.7) ***
Zinc, mg	3.75 (2.38)	7.06 (4.48) ***	4.10 (2.59)	7.69 (4.71) ***	4.80 (2.90)	6.89 (2.90) ***
Choline, mg	164 (162)	184 (162) *	197 (191)	277 (188) ***	206 (188)	223 (175)

DHA, mg	31.1 (48.5)	55.5 (72.0) ***	29.3 (43.6)	46.4 (57.4) **	31.5 (50.4)	37.0 (59.0)

Data presented as median (IQR). Data was analyzed with Wilcoxon's rank-sum tests. Significant differences from children who did not consume dairy are indicated as *** $p < 0.001$, ** $p < 0.01$, * $p < 0.05$.

Table S4. Total daily energy and nutrient intake in children consuming versus not consuming dairy at breakfast stratified per age group in Vietnam using the 24-hour dietary recall.

	**2 to 3 years old**		**4 to 6 years old**		**7 to 12 years old**	
	**Not consumed dairy (n = 493)**	**Consumed dairy (n = 169)**	**Not consumed dairy (n = 563)**	**Consumed dairy (n = 170)**	**Not consumed dairy (n = 1345)**	**Consumed dairy (n = 169)**
Energy, kcal	922 (372)	963 (339)	1060 (474)	1180 (482) **	1220 (544)	1330 (581) **
Protein, g[1]	37.8 (17.6)	40.1 (19.3) *	45.7 (24.6)	48.2 (18.3) *	52.6 (27.9)	58.4 (29.6) *
Carbohydrates, g	138 (57)	136 (53)	162 (74)	169 (62)	184 (86)	182 (79)
Fat, g	22.8 (14.6)	27.3 (14.9) ***	24.8 (19.4)	31.0 (16.1) ***	27.3 (20.4)	36.1 (20.6) ***
Vitamin A, µg RAE	318 (326)	394 (310) ***	263 (316)	370 (271) ***	246 (363)	309 (260) ***
Vitamin B_{12}, µg	1.30 (1.31)	1.51 (1.35) **	1.12 (1.56)	1.62 (1.37) ***	0.96 (1.61)	1.50 (1.30) ***
Vitamin C, mg	19.6 (34.2)	19.7 (34.0)	17.3 (36.0)	16.2 (31.5)	17.1 (43.2)	19.8 (44.3)
Vitamin D, µg	3.37 (5.98)	4.70 (4.57) ***	2.39 (4.51)	4.07 (4.59) ***	1.74 (4.00)	4.25 (4.01) ***
Calcium, mg	423 (301)	536 (256) ***	318 (297)	478 (274) ***	291 (231)	486 (303) ***
Iron, mg	5.42 (3.15)	5.97 (3.59)	6.15 (3.71)	6.52 (3.16)	7.11 (3.89)	7.71 (4.13) *
Zinc, mg[2]	4.60 (2.78)	4.96 (2.57)	5.14 (3.12)	5.51 (2.84) *	5.76 (3.84)	6.11 (3.44)

Data presented as median (IQR). Data was analyzed with Wilcoxon's rank-sum tests. Significant differences from children who did not consume dairy are indicated as *** $p < 0.001$, ** $p < 0.01$, * $p < 0.05$. [1] Outlier removed in children aged 2 to 3 years: Not consumed dairy (n = 482), Consumed dairy (n = 167).

Association analysis between breakfast skipping and stunting and overweight/obesity

Table S5: Multivariate binary logistic regression analysis of factors associated with stunting and overweight/obesity in South-East Asian children stratified by age group and country.

**Outcome and reference**	**Predictor**	**β (SE)**	**95% Confidence interval for odds ratio**		
			**Lower**	**Odds ratio**	**Upper**
**Stratified by Age group**					
**2-6 years old**					
**Stunting**	Intercept	-2.72 (0.16)***	0.05	0.07	0.09
Non-skippers	Semi-skippers	0.15 (0.23)	0.73	1.17	1.79
	Skippers	-0.93 (0.37)*	0.18	0.40	0.76
Urban	Rural	0.14 (0.09)	0.97	1.15	1.38
High income group	Middle income group	0.45 (0.18)*	1.11	1.56	2.23

	Low income group	0.68 (0.17)***	1.43	1.98	2.78
	Poverty	1.19 (0.17)***	2.34	3.28	4.65
**Overweight/obesity**	Intercept	-1.96 (0.12)***	0.11	0.14	0.18
Non-skippers	Semi-skippers	-0.36 (0.30)	0.36	0.70	1.21
	Skippers	-0.05 (0.28)	0.53	0.95	1.59
Female	Male	0.30 (0.09)***	1.13	1.35	1.61
High income group	Middle income group	-0.14 (0.13)	0.67	0.87	1.13
	Low income group	-0.30 (0.13)*	0.58	0.74	0.96
	Poverty	-0.76 (0.16)***	0.34	0.47	0.64
**7-12 years old**					
**Stunting**	Intercept	-3.74 (0.21)***	0.02	0.02	0.04
Non-skippers	Semi-skippers	0.26 (0.20)	0.87	1.30	1.88
	Skippers	0.13 (0.18)	0.79	1.14	1.61
Urban	Rural	0.36 (0.11)**	1.16	1.44	1.79
High income group	Middle income group	0.79 (0.23)***	1.42	2.21	3.56
	Low income group	1.12 (0.22)***	2.01	3.05	4.82
	Poverty	1.77 (0.22)***	3.84	5.85	9.27
**Overweight/obesity**	Intercept	-0.70 (0.08)***	0.42	0.50	0.58
Non-skippers	Semi-skippers	-0.22 (0.14)	0.61	0.80	1.06
	Skippers	0.30 (0.11)**	1.08	1.35	1.69
Female	Male	0.39 (0.06)***	1.30	1.47	1.67
Urban	Rural	-0.18 (0.07)**	0.73	0.83	0.95
High income group	Middle income group	-0.11 (0.09)	0.75	0.90	1.07
	Low income group	-0.44 (0.09)***	0.54	0.64	0.77
	Poverty	-0.82 (0.11)***	0.35	0.44	0.54
**Stratified by country**					
**Malaysia**					
**Stunting**	Intercept	-2.55 (0.17)***	0.06	0.08	0.11
Non-skippers	Semi-skippers	0.03 (0.30)	0.55	1.03	1.78
	Skippers	-0.56 (0.33)	0.29	0.57	1.04
2-6 years old	7-12 years old	-0.50 (0.17)**	0.43	0.61	0.85
High income group	Middle income group	0.14 (0.25)	0.70	1.15	1.87
	Low income group	0.23 (0.21)	0.83	1.26	1.92
	Poverty	0.71 (0.23)**	1.28	2.04	3.21
**Overweight/obesity**	Intercept	-2.53 (0.14)***	0.06	0.08	0.11
Non-skippers	Semi-skippers	0.11 (0.18)	0.78	1.12	1.58
	Skippers	0.60 (0.14)***	1.38	1.83	2.42
2-6 years old	7-12 years old	1.35 (0.12)***	3.03	3.84	4.90
Female	Male	0.27 (0.11)**	1.07	1.32	1.62
High income group	Middle income group	0.12 (0.15)	0.84	1.13	1.52
	Low income group	0.24 (0.13)	0.99	1.28	1.64
	Poverty	0.08 (0.16)	0.78	1.08	1.49

**Thailand**					
**Stunting**	Intercept	-4.43 (0.58)***	0.00	0.01	0.03
Non-skippers	Semi-skippers	-0.42 (0.60)	0.16	0.66	1.79
	Skippers	0.32 (0.53)	0.41	1.37	3.41
High income group	Middle income group	1.36 (0.65)*	1.23	3.88	17.07
	Low income group	1.19 (0.60)*	1.19	3.28	13.55
	Poverty	1.57 (0.60)**	1.72	4.80	19.96
**Overweight/obesity**	Intercept	-1.60 (0.16)***	0.15	0.20	0.27
Non-skippers	Semi-skippers	0.00 (0.24)	0.61	1.00	1.59
	Skippers	0.08 (0.28)	0.62	1.08	1.83
2-6 years old	7-12 years old	1.06 (0.10)***	2.38	2.90	3.54
High income group	Middle income group	-0.07 (0.21)	0.62	0.93	1.40
	Low income group	-0.21 (0.16)	0.59	0.81	1.13
	Poverty	-0.64 (0.18)***	0.37	0.53	0.75
**Indonesia**					
**Stunting**	Intercept	-1.93 (0.48)***	0.05	0.15	0.34
Non-skippers	Semi-skippers	0.31 (0.22)	0.88	1.36	2.06
	Skippers	-0.28 (0.23)	0.47	0.76	1.17
2-6 years old	7-12 years old	-0.42 (0.11)***	0.53	0.66	0.81
Urban	Rural	0.54 (0.11)***	1.39	1.71	2.11
High income group	Middle income group	0.28 (0.50)	0.54	1.32	3.98
	Low income group	0.55 (0.49)	0.73	1.73	5.11
	Poverty	0.88 (0.49)	1.01	2.40	7.10
**Overweight/obesity**	Intercept	-2.54 (0.45)***	0.03	0.08	0.18
Non-skippers	Semi-skippers	-1.45 (0.52)**	0.07	0.24	0.57
	Skippers	0.28 (0.23)	0.83	1.33	2.07
2-6 years old	7-12 years old	1.53 (0.17)***	3.32	4.62	6.54
Urban	Rural	-0.22 (0.15)	0.60	0.81	1.08
High income group	Middle income group	-0.23 (0.46)	0.34	0.79	2.09
	Low income group	-0.43 (0.44)	0.29	0.65	1.68
	Poverty	-0.73 (0.45)	0.21	0.48	1.25
**Vietnam**					
**Stunting**	Intercept	-3.21 (0.27)***	0.02	0.04	0.07
Non-skippers	Semi-skippers	0.35 (0.47)	0.53	1.42	3.38
	Skippers	0.36 (0.43)	0.56	1.43	3.15
2-6 years old	7-12 years old	-0.67 (0.14)***	0.39	0.51	0.67
Urban	Rural	0.38 (0.17)*	1.05	1.46	2.07
High income group	Middle income group	0.68 (0.27)*	1.20	1.97	3.44
	Low income group	1.24 (0.28)***	2.05	3.47	6.18
	Poverty	2.52 (0.31)***	6.95	12.47	23.41
**Overweight/obesity**	Intercept	-1.16 (0.13)***	0.24	0.31	0.41
Non-skippers	Semi-skippers	-0.62 (0.56)	0.15	0.54	1.47

	Skippers	-1.47 (0.61)*	0.05	0.23	0.66
2-6 years old	7-12 years old	1.10 (0.10)***	2.49	3.00	3.62
Female	Male	0.62 (0.09)***	1.55	1.86	2.23
Urban	Rural	-0.65 (0.10)***	0.43	0.52	0.63
High income group	Middle income group	-0.45 (0.11)***	0.51	0.64	0.80
	Low income group	-0.97 (0.15)***	0.28	0.38	0.51
	Poverty	-1.79 (0.35)***	0.08	0.17	0.31

β (SE), regression coefficient (standard error). * P <0.05, ** P <0.01, *** P <0.001.
